# Plasma Proteome Profiles Associated with Inflammation, Angiogenesis, and Cancer

**DOI:** 10.1371/journal.pone.0019721

**Published:** 2011-05-12

**Authors:** Karen S. Kelly-Spratt, Sharon J. Pitteri, Kay E. Gurley, Denny Liggitt, Alice Chin, Jacob Kennedy, Chee-Hong Wong, Qing Zhang, Tina Busald Buson, Hong Wang, Samir M. Hanash, Christopher J. Kemp

**Affiliations:** 1 Division of Human Biology, Fred Hutchinson Cancer Research Center, Seattle, Washington, United States of America; 2 Division of Public Health Sciences, Fred Hutchinson Cancer Research Center, Seattle, Washington, United States of America; 3 Department of Comparative Medicine, University of Washington, Seattle, Washington, United States of America; Institut Jacques Monod, France

## Abstract

Tumor development is accompanied by a complex host systemic response, which includes inflammatory and angiogenic reactions. Both tumor-derived and systemic response proteins are detected in plasma from cancer patients. However, given their non-specific nature, systemic response proteins can confound the detection or diagnosis of neoplasia. Here, we have applied an in-depth quantitative proteomic approach to analyze plasma protein changes in mouse models of subacute irritant-driven inflammation, autoreactive inflammation, and matrix associated angiogenesis and compared results to previously described findings from mouse models of polyoma middle T-driven breast cancer and Pdx1-Cre Kras^G12D^ Ink4a/Arf ^lox/lox^ -induced pancreatic cancer. Among the confounding models, approximately 1/3 of all quantified plasma proteins exhibited a significant change in abundance compared to control mice. Of the proteins that changed in abundance, the majority were unique to each model. Altered proteins included those involved in acute phase response, inflammation, extracellular matrix remodeling, angiogenesis, and TGFβ signaling. Comparison of changes in plasma proteins between the confounder models and the two cancer models revealed proteins that were restricted to the cancer-bearing mice, reflecting the known biology of these tumors. This approach provides a basis for distinguishing between protein changes in plasma that are cancer-related and those that are part of a non-specific host response.

## Introduction

The effective treatment of cancer relies on an accurate diagnosis at the earliest stage of the disease and prognosis is significantly improved if cancer is detected early [1]. Blood-based tests for early detection of cancer are ideal, given that blood draws are inexpensive, minimally invasive, and routine in clinical practice. While the concept of a blood-based cancer test is simple, its application has been challenging, to the point that very few new cancer biomarkers have been FDA approved in recent years [2]. Furthermore, those currently in use, such as CA125 and PSA for ovarian and prostate cancer respectively, are substantially limited by high false positive rates and over-diagnosis [3], [4]. A major hurdle to the development of novel blood based biomarkers has been the technical challenge of interrogating the plasma proteome [5]. Another obstacle has been the choice of case/control comparisons in biomarker discovery [6]. Levels of candidate biomarkers from cancer patients are frequently compared to healthy individuals. In these studies, it is difficult to control for genetic or environmental variables, as well as non-cancerous “confounding” conditions. For example, inflammation and angiogenesis are hallmarks of cancer, but also occur during infections, chronic inflammation, auto-reactive diseases and other conditions that are not specific to cancer [7]. While some biomarker studies have used inflammatory conditions as controls [8], [9], this does not obviate the need to determine the range of proteins that occur with inflammatory conditions. Indeed, the fact that many candidate cancer biomarkers lack sufficient specificity to be useful argues for a new approach [1], [6].

Inflammation and angiogenesis play important roles at all stages of cancer progression, including tumor initiation, growth, and metastatic dissemination. Indeed, chronic inflammatory conditions are strong risk factors for cancer [10]. Inflammation incites and promotes carcinogenesis by causing cell and genome damage, stimulating cellular turnover via cytokines and growth factor release, and creating a tissue microenvironment that can enhance invasion, migration and angiogenesis. Finally, the immune system also controls cancer progression through immunosurveillance and immunoediting [11]. Inflammation is a complex process enacted by the host to control tissue damage against pathogenic, traumatic, or toxic injury and is generally categorized into an acute, rapid response, a sub-acute transition phase, and a persistent but slowly evolving chronic condition. The acute inflammatory response involves a rapid delivery of blood components, plasma, neutrophils, and leukocytes to the site of infection or injury, followed by an influx of macrophages to resolve and repair the injury [12]. Acute phase proteins found in the plasma are defined as either positive or negative depending on whether they increase or decrease during an inflammatory disorder. If an acute inflammatory response fails to resolve the damage, a transition is made to an evolving subacute stage, then to a chronic inflammatory condition. Chronic inflammation, such as that seen in some autoreactive conditions, may be “active”. This is characterized by tissue remodeling, macrophages, T and B cells, angiogenic and other factors. Chronic inflammation can, in turn, lead to excessive tissue damage, immune deregulation, and autoimmunity [10], [13].

Angiogenesis, the sprouting of new blood vessels from preexisting vasculature, is required to provide oxygen and other nutrients to support tumor growth; and the degree of vascularization is a prognostic factor that correlates with tumor aggressiveness [14]. However, angiogenesis is also associated with non-cancerous conditions such as wound healing, tissue repair, rheumatoid arthritis, and other chronic inflammatory diseases [15]. Thus, inflammation and angiogenesis are intimately involved in cancer as well as other common pathologies. These conditions trigger complex and dynamic systemic responses, but the extent of overlap between the systemic response to cancer and to non-cancerous conditions is largely unknown.

To characterize the systemic response to cancer and inflammation, we applied plasma proteomics to mouse models of subacute inflammation, chronic inflammation, and angiogenesis. Mouse models overcome some key hurdles for biomarker discovery, including technology development [16]; more precise matching of cases and controls to reduce genetic, age, and environmental variables; interrogating the dynamic changes in the plasma proteome through disease progression; and integrating results with the extensive biological knowledge bases [17]. Carrageenan injections were used to model subacute inflammation [18]. This model represents a subcuticular response of evolving local irritation and necrosis similar to what might be seen following tumor necrosis. For chronic inflammation, we utilized a collagen-induced model of arthritis [19]. This model is associated with antigen-antibody complexes, consistent with an auto-reactive lesion, with infiltration of mononuclear cells, bone remodeling, fibroplasia and vascular growth surrounding the joint. FGF injections were used to stimulate angiogenesis, resulting in rapid in-growth of blood vessels and supporting stromal elements [20].

A large number of plasma proteins changed in abundance in each model, reflecting the complex biology of each condition. These plasma profiles were compared to those previously identified in two well characterized mouse models of pancreatic [21] and breast cancer [22]. In the breast cancer model, the oncoprotein polyoma middle T antigen (PyMT) is driven by the MMTV LTR and is restricted to the mammary epithelium. These mice develop carcinomas that resemble human breast cancer and that are associated with leukocyte infiltration and subsequent pulmonary metastasis [23]. Development of pancreatic cancer in the Pdx1-Cre Kras^G12D^ Ink4a/Arf^lox/lox^ model involves progression from pancreatic intraepithelial neoplasia (PanINs) to pancreatic ductal adenocarcinoma (PDAC), faithfully recapitulating the human disease [24]. Here we show that the majority of plasma protein changes identified in these tumor models were unique to each model and not seen in the confounder models. Furthermore, many of these proteins have known roles in cancer progression. The identification and characterization of protein profiles associated with cancer versus non-cancerous pathologies can be used to understand the complex biology of the host response to cancer and to prioritize candidate biomarkers that are associated with cancer.

## Materials and Methods

### Animal studies

Animal studies were performed under IACUC regulations as approved by the FHCRC animal use committee (Protocol #1311). Ten female FVB mice were used for each condition paired with ten littermate untreated controls. To model acute inflammation that transitions into subacute inflammation, we used a well known pro-inflammatory irritant, carrageenan, a carbohydrate derived from seaweed [18]. This was delivered via a sponge implant which sustains the carrageenan release and contributes a classical foreign body response. 10×10 mm surgical sponges were injected with 1% carrageenan (Sigma C1867-5G) and implanted subcutaneously into the right flank. Plasma was collected by cardiac puncture 3 weeks later. The plasma proteins identified from these mice should correspond to a late stage subacute response to the carrageenan and associated sponge impact, rather than to initiation of the acute inflammatory response which occurs within 72 hours. To evaluate the protein profile associated with chronic inflammation, we used a collagen-induced arthritis mouse model [19]. Bovine collagen type II (CII) (BD Biosciences, San Jose, CA) was emulsified with complete Freund's adjuvant (Pierce, Rockford, IL) at a final concentration of 4 mg/ml and a total of 0.1 ml was injected intradermally near the base of the tail. This results in the development of chronic arthritis in the hind paws within 14–21 days. Mice were monitored every 2–3 days for the development and progression of arthritis and plasma collected upon development of swollen hind paws at 4 weeks. To model angiogenesis, matrigel (BD Biosciences, San Jose, CA) plus FGF (500 ng/ml) (Invitrogen, Carlsbad, CA) was injected subcutaneously into the right flank resulting in rapid in-growth of blood vessels and supporting stromal elements but with little associated inflammation [20]. Plasma was collected 3 weeks later. For these models, blood from experimental and control mice was collected by cardiac puncture, using a 1 cm^3^ syringe and a 23 g needle, and placed in a K3-EDTA tube (Webster Veterinary Supply, Sterling, MA) [17]. Plasma was isolated by centrifugation at 2000×g for 5 min and aliquots transferred to cryovials and frozen at −80°C.

Sample collection for the pancreatic [21] and breast [22] cancer mouse models has been previously described. Briefly, mice for pancreatic cancer proteomic analysis were obtained by breeding Pdx1-Cre *Ink4a/Arf* ^lox/lox^ and *Kras* ^G12D^ *Ink4a/Arf* ^lox/lox^ mice. Experimental Pdx1-Cre *Kras* ^G12D^ *Ink4a/Arf* ^lox/lox^ mice and control *Kras* ^G12D^ *Ink4a/Arf* ^lox/lox^ and Pdx1-Cre *Ink4a/Arf* ^lox/lox^ mice were sacrificed at 5.5 and 7 weeks of age. For both case and control mice, lethal comas were induced by injecting mice i.p. with a 0.6–0.8 mL 5% Avertin (2,2,2-Tribromoethanol, Sigma). This difference in euthanasia method introduces a potential, although likely minor, caveat when comparing the pancreas models to the other models. A 1-mL syringe with a 22 g needle was used for cardiac puncture to obtain blood. Blood was placed in K3-EDTA tubes (Fisher) and centrifuged at 4°C for 5 min at 3000 rpm. Plasma was removed and stored at −80°C.

For the breast cancer mouse model, transgenic FVB/N-Tg(MMTV-PyVT)634Mul/J (PyMT) mice were obtained from the National Cancer Institute and bred in-house to obtain plasma samples from tumor-bearing mice and control littermates at two time points of breast cancer development. PyMT heterozygote males were crossed to FVB wild-type females to generate the cohort of PyMT heterozygote and wild-type females for study. To avoid bias, PyMT transgenic and control mice were paired at weaning and were matched with respect to age, litter, and cage. All mice were fed standard chow (Harland Tekland, 8664) and acidified water *ad libitum* and kept on a 12 h light–dark cycle. Beginning at 5 weeks of age, mice were palpated every other day to detect breast tumor growth. Breast tumors were allowed to develop to either 0.5 or 1 cm in diameter, after which each tumor-bearing mouse and a control were euthanized back-to-back on the same day by CO_2_ inhalation. Blood was obtained by cardiac puncture and plasma was isolated and stored as described for the inflammation and angiogenesis mouse models.

### Immunodepletion of abundant proteins and isotopic labeling

50 µl of plasma from 5 mice was pooled for each set of case and control samples. Pools were immunodepleted of the top three most abundant proteins (albumin, IgG, and transferrin) using a MS-3 column (4.6×10 mm, Agilent). The immunodepletion column was equilibrated with buffer A at (0.5 ml/min) for 13 min and aliquots of pooled sera were injected after filtration through a 0.22 µm syringe filter. The flow-through fractions were collected for 10 min at a flow rate of buffer A of 0.5 mL/min, combined and stored at −80°C. The column bound material was recovered by elution for 8 min with buffer B at 1 ml/min. Subsequently, immunodepleted samples were concentrated using Centricon YM-3 devices (Millipore Corp, Bedford, MA) and re-diluted in 8 M urea, 100 mM Tris pH 8.5, 0.5% OG (octyl-beta-d-glucopyranoside, Roche). Following depletion and buffer exchange, protein concentrations were determined by Bradford assays: angiogenesis 2.5 mg (case) and 3.8 mg (control), chronic inflammation 4.3 mg (case) and 3.8 mg (control), and acute inflammation 2.8 mg (case) and 3.1 mg (control), and the entire amount of protein for each sample was used for subsequent acrylamide labeling. Samples were reduced with DTT (0.66 mg DTT/mg protein), and isotopic labeling of cysteine residues on intact proteins was performed. Control samples were labeled with light 12C acrylamide isotope (7.1 mg/mg protein) (>99.5% purity, Fluka), and experimental samples were labeled with the heavy 13C-acrylamide isotope (Cambridge Isotope Laboratories, 7.4 mg/mg protein) for 1 h at room temperature. Pools of control and experimental samples were then combined for intact protein separation.

### Intact protein separation

The combined isotopically labeled samples were separated by an automated online 2D-HPLC system controlled by Workstation Class-VP 7.4 (Shimadzu Corporation). The combined labeled plasma samples were separated in the first dimension on an anion exchange column (Poros HQ/10, 10 mmID×100 mL, Applied Biosystems) using an 8 step-elution (from 0 mM NaCl to 1000 mM NaCl) at 0.8 mL/min. Fractions from each of the 8 anion-exchange separation elution steps were automatically transferred onto a reversed-phase column (PorosR2/10, 4.6 mm ID×100 mL, Applied Biosystems) for a second dimension of separation. A 25 min gradient elution (from 5% to 95% mobile phase B) was used at 2.4 mL/min. 3 fractions were collected per minute, with each fraction containing 800 uL. 576 reversed phase fractions were collected in total (8 anion exchange fractions each separated into 72 reversed phase fractions). Mobile phase A for anion-exchange chromatography consisted of 20 mM Tris (Sigma), 6% isopropanol (Fisher), 4 M urea, pH 8.5 and mobile phase B was the same composition and pH as A with 1 M NaCl (Fisher) added. Mobile phase A for reversed-phase chromatography consisted of 95% water, 5% acetonitrile, 0.1% TFA (Supelco), and mobile phase B consisted of 90% acetonitrile, 10% water, 0.1% TFA.

### Mass spectrometry analysis

In-solution digestion was performed with lyophilized aliquots from the reversed-phase (second dimension) fractionation step. Proteins in individual fractions were re-suspended in 0.25 M urea (Fisher) containing 50 mM ammonium bicarbonate and 4% acetonitrile and then digested overnight with 200 ng of modified trypsin (Promega) at 37°C. The resulting peptide mixtures were acidified with 5 µL of 1% formic acid. Aliquots were subjected to mass spectrometry shotgun analysis after pooling of several consecutive fractions (protein concentration estimated by UV trace). 12 pools of individual reversed-phase fractions for each of the 8 anion exchange steps were created by combining the following sequential fractions: 1–22, 23–24, 25–26, 27–28, 29–30, 31–32, 33–34, 35–36, 37–38, 39–40, 41–42, and 43–72. 96 fractions were analyzed for each experiment by a LTQ-Orbitrap (Thermo) mass spectrometer coupled with a NanoLC-1D (Eksigent). The liquid chromatography separation was performed in a 25 cm column (Picofrit 75 µm i.d., New Objectives, packed in-house with MagicC18 resin) using a 90 min linear gradient from 5 to 40% of acetonitrile in 0.1% formic acid at 300 nl/min for shotgun analysis. Approximately 10 ug of protein was injected per fraction. Spectra were acquired in a data-dependent mode in *m*/*z* range of 400–1800, including selection of the 5 most abundant +2 or +3 ions of each MS spectrum for MS/MS analysis. Mass spectrometer parameters were capillary voltage of 2.0 KV, capillary temperature of 200°C, resolution of 60,000, and target value of 1,000,000.

### Data processing

Acquired data were automatically processed by the Computational Proteomics Analysis System (CPAS) [25] pipeline using the X!Tandem search algorithm [26] configured with comet score module plug-in. PeptideProphet [27] and ProteinProphet [28] were used for validation of search results and protein inference. Quantitation was performed using the Q3 quantitation tool [29]. The tandem mass spectra were searched against version 3.48 of the mouse IPI database [30]. All identifications with a PeptideProphet probability greater than 0.2 were submitted to ProteinProphet, and the subsequent protein identifications were filtered at a minimum 5% error rate. For quantitation, ratios for proteins were computed using only those peptides achieving PeptideProphet probability of at least 0.75. Protein groups assigned by ProteinProphet were combined by common gene symbol and henceforth referred to as “protein”. Case/control ratios for each protein were computed by averaging log2 ratios across all peptides assigned to the protein. Over 1,000,000 MS/MS spectra were collected and proteins with 2289 unique gene names were identified across the 3 experimental models.

Statistical significance of protein quantification by mass spectrometry was determined by two methods. For proteins with multiple paired MS events of heavy and light acrylamide, a one-sample t-test was used to calculate a p-value for the mean ratio of the whole protein across all fractions. Second, the probability for the ratio for each MS event was calculated from the distribution of ratios in a control-control experiment in which the same sample was split and labeled with heavy and light acrylamide. If the p-value for each individual event was <0.05, the protein ratio was considered statistically significant.

### Network analysis

The increased and decreased proteins identified from the IPAS analysis were used for biofunction and pathway analysis using Ingenuity Pathway Analysis (IPA) Software (Ingenuity Systems, Mountain View, CA). The IPA database consists of proprietary ontology representing 300,000 biologic genes, proteins, and molecular and cellular processes.

### Enzyme linked immunosorbent (ELISA) assays

Plasma levels of Pf4, Igf1, Igfbp5, and Lcn2 were measured using commercially available mouse DuoSet kits obtained from R&D Systems (Minneapolis, MN). Plasma was diluted 1∶1000, 1∶400, 1∶120, and 1∶180 for Pf4, Igf1, Igfbp5, and Lcn2, respectively, for testing. Assays were performed according to the manufacturer's protocol and samples were assayed in duplicate.

## Results

### Identification of plasma proteins distinct to tumor-bearing mice

**Figure 1:**
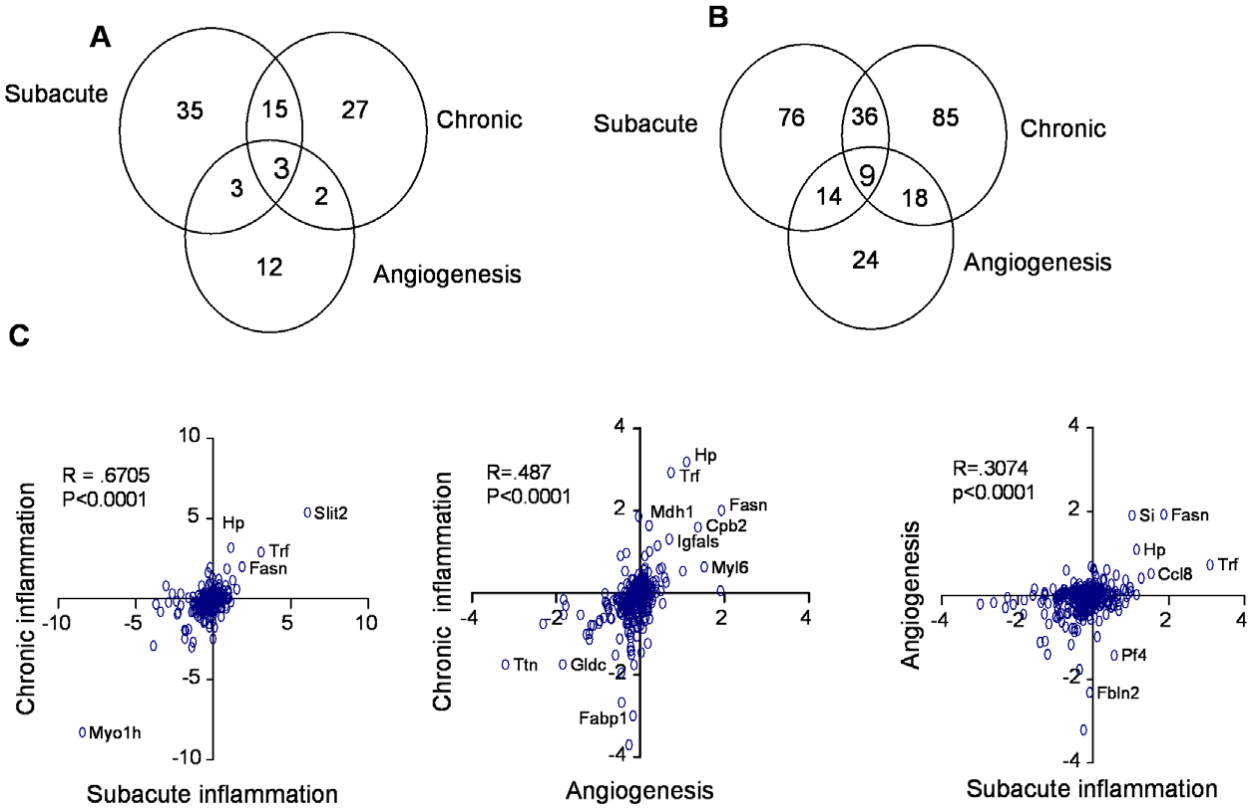
Comparison of plasma proteomic changes in confounding models. Venn diagrams comparing (A) increased and (B) decreased proteins in plasma from the subacute and chronic inflammation and angiogenesis models as compared to control mice. Diagrams show numbers of proteins, either elevated or reduced in each model, and which are unique or shared between each of the 3 models. The majority of either increased or decreased proteins were unique to each model. (C) Correlation plots for quantified proteins. Case/control (log2) ratios for each quantified protein are plotted on the X- and Y- axes. The plots reflect abundance differences of specific proteins between two models. Proteomic comparisons between chronic versus subacute inflammation comparison are more similar (R = .67) than are comparisons between either inflammation model and the angiogenesis model (R = .49 and .31, respectively).

To identify cancer-restricted plasma proteins, we compared the plasma proteomes of mice with carageenan-induced subacute inflammation, collagen-induced arthritis, and FGF-induced angiogenesis to the plasma proteomes of mice with PyMT driven breast cancer and Pdx1-Cre Kras^G12D^ Ink4a/Arf ^lox/lox^ pancreatic cancer. Plasmas obtained from mice with subacute inflammation, chronic inflammation, and angiogenesis, along with aged-matched control mice were subjected to in-depth proteomic analysis. In proteomic comparisons of plasmas from mice with confounding condition to control mice, between 378 to 511 proteins were quantified based on differential isotopic labeling on cysteine residues (Table S1). Variability in the number of quantified proteins reflects variability in protein measurement and mass spectrometry sampling. Remarkably, approximately one third of all quantified proteins changed in abundance by 1.25-fold or greater compared to control mice (p<0.05) and, of these, two to three times as many were decreased as opposed to increased in all three models (Table 1, Figure S1). When we consider only proteins quantified in all three mouse models, comparisons of plasma profiles between the models revealed a 35% overlap in altered proteins between subacute and chronic inflammation models, compared to only a 15% overlap between the inflammation models and the angiogenesis model (Table 1). Due to the limited sampling of the mass spectrometer, a number of proteins were not quantified in all three mouse models. When we do not require proteins to be quantified in all three mouse models, the overlap of up- and down-regulated proteins is shown in Figure 1A and 1B respectively. Comparisons of changes in protein levels for each model revealed a strong correlation between subacute and chronic inflammation, with a Pearson test score of 0.67 (Figure 1C), while comparisons of each inflammation model to the angiogenesis model revealed less than 50% correlations (Pearson test scores of 0.49 and 0.31, respectively). Thus, plasma profiles were more similar between inflammation models than between angiogenesis and either inflammation model, reflecting the underlying biology of these conditions. Further, the majority of altered proteins were unique to each confounder model, demonstrating biological specificity. The relative abundances of the individual proteins identified in each of the three models are listed in Table S1.

**Table 1:** Comparison of proteins identified in plasma from the 3 mouse models.

	Number of proteins*			
	Identified	Quantified	Increased**	Decreased**
**Acute inflammation**	1611	411	56	135
**Chronic inflammation**	1706	511	47	148
**Angiogenesis**	1255	378	20	65

*Unique gene names.

**Case compared to control (1.25-fold or greater, p-value<0.05).

**Figure 2:**
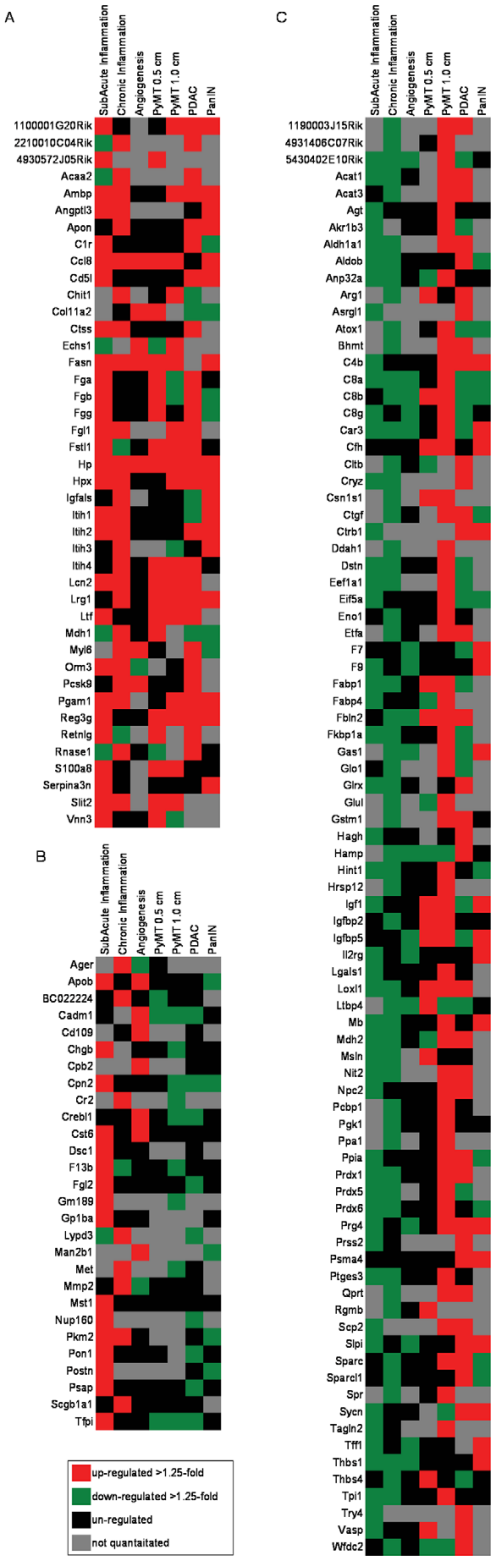
Heat map of plasma proteins altered in both confounders and cancer models. (A) Proteins increased in both confounders and cancer models compared to controls (>1.25 fold, p<0.05), (B) proteins increased in confounders and decreased in cancer models, and (C) proteins decreased in confounders and increased in cancer models (red = up, green = down, black = no change, grey = not quantified).

**Figure 3:**
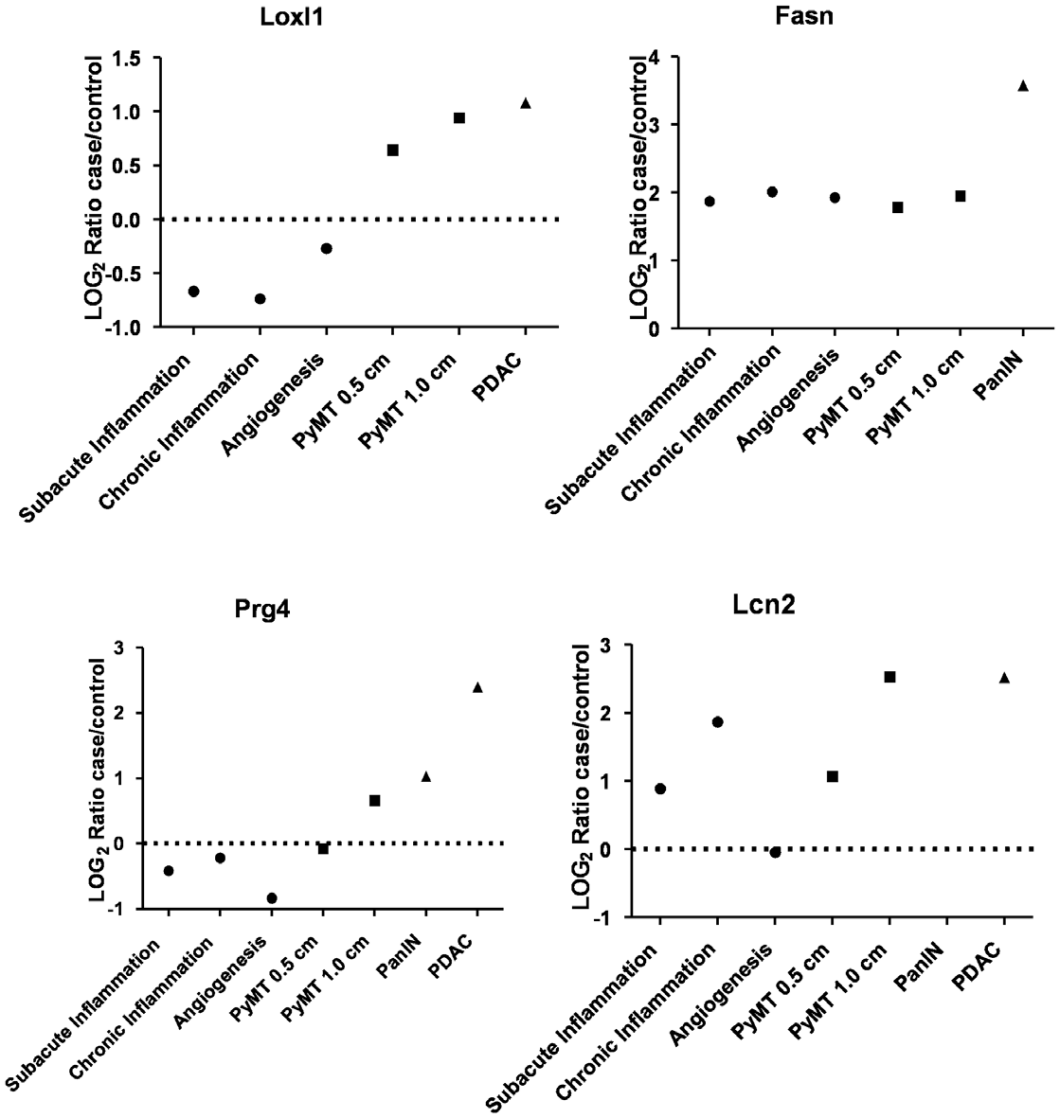
Comparisons of selected proteins in plasma from confounders and cancer models. Plots show mass spectrometry based log2 case/control ratios for specific proteins in each confounder and cancer model that display differential abundance patterns. Loxl1 represents proteins that are reduced in the confounders and elevated in the cancer models. Fasn shows elevation in all models but more so in PanIN. Prg4 is elevated primarily in the pancreatic cancer model. Lcn2 is elevated in the inflammation and to a greater degree in the cancer models, but not the angiogenesis model.

We then compared the proteomic profiles of these confounding models to previously obtained profiles from early and late stage breast cancer [22], and to profiles from early stage (PanINs) and late stage (PDAC) pancreatic cancer [21]. In contrast to the confounder models, a roughly equal number of proteins were increased and decreased in tumor-bearing mice compared to non-tumor-bearing mice (Table 2). Of these altered proteins, the great majority (>75%) were not altered in confounders (Table 2). Three patterns of plasma protein distribution were observed: increased in both the confounders and the cancer models (Figure 2A), increased in confounders but unchanged or decreased in cancer (Figure 2B), and decreased in confounders and increased in cancer (Figure 2C). Only 27% of proteins with altered levels in the study were increased in both confounders and cancer, while the largest category, accounting for 55% of the total, consisted of proteins that were decreased in confounders but increased in tumor-bearing mice. Relative levels of representative proteins displaying these patterns are shown in Figure 3. Note, lysyl oxidase like 1 (Loxl1) and proteoglycan 4 (Prg4) are reduced in plasma from confounders and increased in both breast and pancreatic tumor-bearing mice, while fatty acid synthase (Fasn) and lipocalin2 (Lcn2) are increased in both confounders and cancer models (Table S1).

**Table 2:** Comparison of quantified proteins from confounding and tumor models.

			Acute	Chronic	Angiogenesis
			inflammation	inflammation	
Tumor model	****	# Proteins*	# Proteins shared with tumor models		
**PyMT 0.5 cm** **	**Up**	94	13	6	4
	**Down**	111	12	11	5
**PyMT 1.0 cm** **	**Up**	192	13	11	4
	**Down**	127	6	13	7
**PanIN** ***	**Up**	89	11	11	3
	**Down**	129	17	18	6
**PDAC** ***	**Up**	141	15	13	1
	**Down**	194	26	21	17

*Unique gene names.

**As reported in J. Proteome Res. 2008, 7, 1481–1489.

***As reported in PLoS Med. 2008, 5, e123.

****Up and down refers to case compared to control (1.25-fold or greater, p-value<0.05).

**Figure 4:**
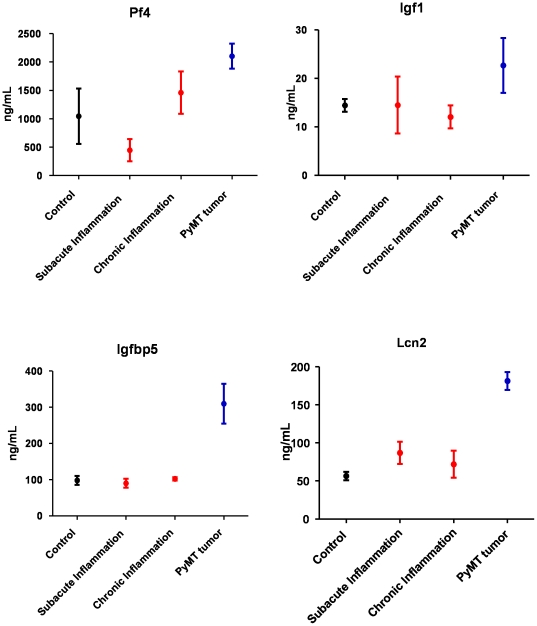
Quantitative analysis of selected proteins increased in cancer and inflammation mouse models. ELISA analysis of Pf4, Igf1, Igfbp5, and Lcn2 showing increased plasma concentration in PyMT breast tumor-bearing mice compared to either the subacute or chronic inflammation mice. Plasma from untreated mice was used as the control.

To verify the differential abundance observed by mass spectrometry, ELISA assays were performed on selected proteins based on commercial availability (Pf4, Igf1, Igfbp5, and Lcn2) (Figure 4). Note, insulin like growth factor 1 (Igf1) and insulin like growth factor binding protein 5 (Igfbp5) were decreased or unchanged in confounders but increased in breast tumor-bearing mice. Lipocalin 2 (Lcn2) was increased in all models but to a higher level in tumor-bearing mice, while platelet factor 4 (Pf4) was decreased in subacute inflammation but increased in chronic inflammation and cancer. Other plasma proteins that were specifically increased in tumor-bearing mice included acetyl-CoA acetyltransferase 1 and 3, fatty acid binding proteins 1 and 4, fibulin, peroxiredoxins 1, 5 and 6, SPARC, and thrombospondins 1 and 4.

### Plasma protein changes observed with inflammation

**Figure 5:**
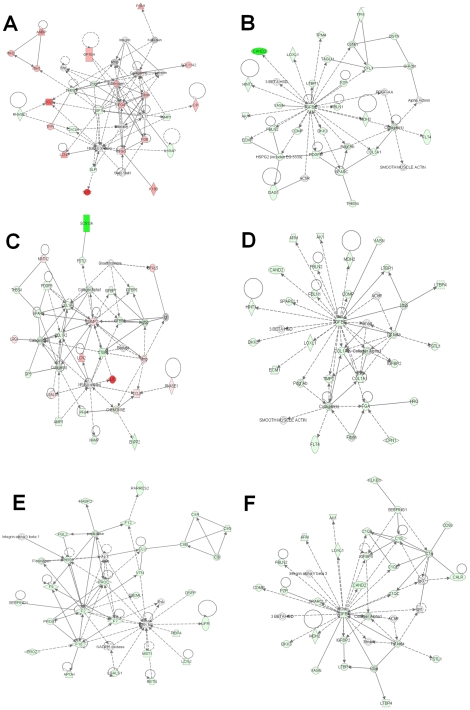
Network analysis of plasma proteins from confounding conditions. Top networks assigned by Ingenuity Pathway Analysis for both increased and decreased proteins from the subacute inflammation (A, B), chronic inflammation (C, D) and angiogenesis models (E, F). Networks for the subacute model show abundant fibrinogen and ECM proteins, the chronic model gives prominent growth factor and collagen networks, while the angiogenesis network shows chemokine and coagulation proteins. [red = increased, green = decreased, white = no change in abundance in cases vs control mice, >1.25 fold p<0.05.].

We next analyzed the altered plasma proteins from each confounder condition. In the subacute inflammation model, greater than 50% of the increased proteins play a known role in inflammation, including positive acute phase proteins [31], as well as the chemokines Ccl8 and Pf4 (also known as Cxcl4) (Table S1). Among the decreased proteins were five complement proteins, as well as secreted signaling proteins such as Rbp4, Igf1, Igf2, TGFβ, and Pdgfb. The complement cascade is important in the acute phase response, while Igf1 and Rb4 are negative acute phase proteins. Network analysis linked many of the proteins with altered levels to intracellular (NF-κB) and secreted (Il-1 and TGFβ) immune system regulators (Figure 5A, B) [10]. TGFβ itself was reduced, as were multiple proteins involved in TGFβ signaling. Collectively, proteomic analysis of plasma identified known acute phase proteins, proteins linked to inflammatory regulators such as TGFβ, and additional proteins (e.g. cytoskeletal proteins actins, cofilins, vasp, profilin, and destrin) not previously linked to inflammation (Table S1).

As in the subacute inflammation model, a significant number of proteins increased under chronic inflammation had known links to inflammation. Notably, however, fewer acute phase response proteins were identified than with the subacute model (Table S1). Another major category of proteins altered with chronic inflammation are involved in extracellular matrix remodeling (Figure 5C). Many of these were decreased and are targets of the Mmp2 protease, which was itself increased. As in the subacute model, TGFβ and proteins linked to its regulation or signaling were reduced, including Ltbp1, Ltbp4, and Vasn (Figure 5D).

### Plasma protein signature for angiogenesis

A significant number of the altered proteins identified in the angiogenic model have known mechanistic links to angiogenesis and/or are expressed in endothelial cells, such as thrombospondin 1 and 4, renin 1 and 2, platelet factor 4, trefoil factor 1, and collagen binding protein 2, as well as other proteins not previously associated with angiogenesis. In addition, alterations in complement proteins and coagulation factors, which have links to angiogenesis, were observed (Figure 5E) [32] [33]. Many proteins involved in TGFβ signaling, including TGFβ itself, were reduced (Figure 5F).

Only three proteins were increased in all three models: Hp, Ccl8, and Fasn, all of which are known to be activated and increased during inflammatory responses [34]–[36]. There were ten decreased proteins shared among the models: C8a, Car3, Ddt, Ltbp1, Mug1, Notch1, Proz, Rbp4, Tagln, and TGFβ. Interestingly, at least four of these are mechanistically linked, in that Ltbp1 and Tagln are involved in the regulation of TGFβ [37] and there is cross talk between Notch1 and TGFβ signaling [38].

## Discussion

Successful development of plasma-based biomarkers for early detection of cancer requires that the markers have both high specificity and sensitivity. In particular, any marker must discriminate between non-cancerous conditions and cancer. Many cancer biomarkers discovered to date lack sufficient specificity and cannot adequately distinguish cancer from confounding conditions [6]. Here, we demonstrate a strategy to identify cancer-associated plasma proteins by controlling for confounding factors. By applying state-of-art proteomic analysis to well defined mouse models, we have identified and quantified approximately 500 plasma proteins associated with subacute inflammation, chronic inflammation, and angiogenesis. Approximately one third of all quantified proteins from these confounder models showed a significant change in abundance, indicating that a significant fraction of the plasma proteome changes during a systemic response. The majority of proteins that changed in abundance were unique to each condition and these accurately reflect the complex underlying biology.

Changes in the plasma proteome from the confounding condition models were compared to the plasma proteomes from two mouse cancer models. Overall, approximately 25% of the proteins altered in the confounder mice were also altered in the tumor bearing mice, reflecting the central roles of both inflammation and angiogenesis in tumor development. In particular, proteins of the fibrinogen cascade, ITIH trypsin inhibitors, haptoglobin and hemopexin predominated across models. These proteins are less likely to be cancer-specific and this list can be used to prioritize cancer associated proteins. For example, lipocalin 2 (Lcn2), a small extracellular protein involved in iron transport, has been shown to promote breast cancer progression and metastasis and is found in urine of patients with metastatic breast cancer [39]. Based on these findings, Yang *et al.* have proposed that Lcn2 may be a biomarker for breast cancer [40]. However, Lcn2 plasma protein levels were elevated under both subacute and chronic inflammation, which may limit its usefulness as a cancer-specific biomarker. Haptoglobin (Hp) has also been suggested as a biomarker for ovarian cancer [41] but we observed elevated levels of Hp in all confounder models. It should be pointed out that our mass spectrometry analysis does not necessarily distinguish between specific isoforms of the same protein and we cannot rule out the presence of “cancer-specific” isoforms of proteins such as haptoglobin.

The majority of the proteins altered in plasma from the tumor bearing mice did not overlap with the three confounding models. This suggests that most of the alterations in the plasma proteome found in tumor bearing mice are specifically associated with cancer. A caveat to this conclusion is that cancer-associated inflammation may significantly differ from the models of inflammation we used here. Also, inflammation associated with early stage cancer is likely to differ from that associated with late stage disease. Of the proteins that differed between confounder and cancer models, the largest category consisted of proteins that were decreased in confounders and increased in cancer. An examination of the proteins in this category reveals many with known associations to breast or pancreas cancer biology, but most have not been reported as altered in plasma. For example, alpha-S1-casein (Csn1s1) is a breast-specific milk protein. Lysyl oxidase (Loxl1), a matrix remodeling protein, is known to play a role in breast cancer migration, adhesion, and metastasis [42]. Insulin like growth factor-1 (Igf-1) signaling promotes proliferation and inhibits apoptosis of breast cancer cells, and high plasma levels of Igf-1 are associated with risk of breast, prostate and other cancers [43]. Insulin like growth factor binding protein 5 (Igfbp5) plays multiple roles in breast cancer progression and is a prognostic factor [44]. Thrombospondin-1 (Thps1), a potent inhibitor of angiogenesis, is associated with breast cancer metastasis [45]. Fibulin 2, an extracellular matrix and plasma glycoprotein, facilitates invasion and migration of breast cancer cells [46]. SPARC, a secreted protein involved in cell/matrix interactions, is overexpressed in pancreatic cancer [47]. Other proteins increased in tumor-bearing mice include fatty acid binding proteins 1 and 4, acetyl-CoA acetyltransferase 1 and 3, and peroxiredoxins 1, 5 and 6. This analysis shows that controlling for confounding conditions can be used to identify and prioritize plasma proteins whose abundance changes with cancer. Future studies will be required to determine the role of these proteins in the plasma with respect to cancer biology and to validate these as cancer specific biomarkers.

Proteomic analysis of plasma also provides biologic insight into the systemic response to inflammation and angiogenic stimuli. Many of the altered proteins from each confounder model grouped into functional categories that are known to participate in the systemic response to these conditions, as well as novel inflammation or angiogenesis-related proteins. For example, in the subacute inflammation model, alterations in plasma proteins were detected from all major functional categories of the acute phase response, including complement system, coagulation, and fibrinolytic systems, antiproteases, transport proteins, and other participants in the inflammatory response [31]. This is consistent with the complex and rapidly evolving changes present during this stage of an irritant and foreign body driven inflammatory process. Altered levels of chemokines, as well as proteins with links to immune regulators such as TGFβ were also seen. The most increased protein identified was Slit-2, a secreted protein that modulates inflammation and inhibits neutrophil migration [48]. Slit, through binding to its receptor Robo inhibits Rho GTPase, which may contribute to the decrease in a number of cytoskeletal proteins seen during inflammation. The chronic inflammation plasma proteome was also dominated by inflammatory proteins but many differed from the subacute model, indicating distinct mechanisms of action and systemic responses to these inflammatory conditions. As expected, a number of altered proteins identified have links to arthritis, including Mmp-2, TGFβ, Fstl1, and Hp [49]. The altered proteins seen in the angiogenesis model included those involved in angiogenesis or vascular and connective tissue biology, including complement and coagulation factors.

A number of proteins identified in all three confounding conditions are involved in TGFβ signaling networks with direct links to the regulators Ltbp1 and 4. TGFβ is a secreted cytokine involved in many cellular processes, including cell proliferation, extracellular matrix remodeling, cell migration, adhesion, invasion, and metastasis [50]. TGFβ is known to play dual roles in the inflammatory response, both inhibitory and promoting, and is also linked to vascular biology [37]. The opposing roles for TGFβ are likely controlled by its selective production and latency, in addition to receptor modulation and differential susceptibility of target cells at various stages of development. Previous studies have shown that latent circulating TGFβ may have protective properties against inflammation. Indeed, systemic administration of TGFβ alleviates the inflammatory response in a mouse model of arthritis [51] and impaired TGFβ signaling during infection with E. coli enhanced peritoneal inflammation in a rat model [52]. That TGFβ and a number of its interacting proteins are altered in plasma from all three models confirms the central role of TGFβ in inflammation and angiogenesis. However, some TGFβ-related proteins exhibited model specificity implying that the TGFβ response is tailored to each condition.

In summary, by applying plasma proteomics to mouse models of disease, we identified novel proteins and protein networks that are involved in the systemic response to both cancer and non-cancerous conditions. By identifying proteins involved in the systemic response of non-cancerous conditions, the specificity of potential cancer biomarkers can be better assessed.

## Supporting Information

Figure S1 **Histograms of case/control peptide ratios.** A) acute inflammation, B) chronic inflammation, and C) angiogenesis.
(PDF)

Table S1 **Case/control ratios for all proteins and peptide identification information are shown for acute inflammation, chronic inflammation, and angiogenesis.**
(XLSX)

## References

[1] Etzioni R, Urban N, Ramsey S, McIntosh M, Schwartz S (2003). The case for early detection.. Nat Rev Cancer.

[2] Gutman S, Kessler LG (2006). The US Food and Drug Administration perspective on cancer biomarker development.. Nat Rev Cancer.

[3] Bell R, Petticrew M, Sheldon T (1998). The performance of screening tests for ovarian cancer: results of a systematic review.. Br J Obstet Gynaecol.

[4] Etzioni R, Penson DF, Legler JM, di Tomaso D, Boer R (2002). Overdiagnosis due to prostate-specific antigen screening: lessons from U.S. prostate cancer incidence trends.. J Natl Cancer Inst.

[5] Jacobs JM, Adkins JN, Qian WJ, Liu T, Shen Y (2005). Utilizing human blood plasma for proteomic biomarker discovery.. J Proteome Res.

[6] Chechlinska M, Kowalewska M, Nowak R (2010). Systemic inflammation as a confounding factor in cancer biomarker discovery and validation.. Nat Rev Cancer.

[7] Karin M, Lawrence T, Nizet V (2006). Innate immunity gone awry: linking microbial infections to chronic inflammation and cancer.. Cell.

[8] Somiari SB, Shriver. CD, Heckman C, Olsen C, Hu H (2006). Plasma concentration and activity of matrix metalloproteinase 2 and 9 in patients with breast disease, breast cancer and at risk of developing breast cancer.. Cancer Letters.

[9] Shaw CA, Lowe KA, Paley P, Wallace E, Anderson GL (2009). Influence of ovarian cancer risk status on the diagnostic performance of the serum biomarkers mesothelin, HE4, and CA125.. Cancer Epidemiol Biomarkers Prev.

[10] Coussens LM, Werb Z (2002). Inflammation and cancer.. Nature.

[11] Dunn GP, Bruce AT, Ikeda H, Old LJ, Schreiber RD (2002). Cancer immunoediting: from immunosurveillance to tumor escape.. Nat Immunol.

[12] Serhan CN, Savill J (2005). Resolution of inflammation: the beginning programs the end.. Nat Immunol.

[13] Nathan C, Ding A (2010). Nonresolving inflammation.. Cell.

[14] Maeda K, Chung YS, Takatsuka S, Ogawa Y, Sawada T (1995). Tumor angiogenesis as a predictor of recurrence in gastric carcinoma.. J Clin Oncol.

[15] Costa C, Incio J, Soares R (2007). Angiogenesis and chronic inflammation: cause or consequence?. Angiogenesis.

[16] Whiteaker J, Zhang H, Zhao L, Wang P, Kelly-Spratt KS (2007). Integrated pipeline for mass spectrometry-based discovery and confirmation of biomarkers demonstrated in a mouse model of breast cancer.. Journal of Proteome Research.

[17] Kelly-Spratt KS, Kasarda AE, Igra M, Kemp CJ (2008). A mouse model repository for cancer biomarker discovery.. J Proteome Res.

[18] Maiuri MC, Tajana G, Iuvone T, De Stefano D, Mele G (2004). Nuclear factor-kappaB regulates inflammatory cell apoptosis and phagocytosis in rat carrageenin-sponge implant model.. Am J Pathol.

[19] Trentham DE, Townes AS, Kang AH (1977). Autoimmunity to type II collagen an experimental model of arthritis.. J Exp Med.

[20] Akhtar N, Dickerson EB, Auerbach R (2002). The sponge/Matrigel angiogenesis assay.. Angiogenesis.

[21] Faca VM, Song KS, Wang H, Zhang Q, Krasnoselsky AL (2008). A mouse to human search for plasma proteome changes associated with pancreatic tumor development.. PLoS Med.

[22] Pitteri SJ, Faca VM, Kelly-Spratt KS, Kasarda AE, Wang H (2008). Plasma proteome profiling of a mouse model of breast cancer identifies a set of up-regulated proteins in common with human breast cancer cells.. J Proteome Res.

[23] Lin EY, Jones JG, Li P, Zhu L, Whitney KD (2003). Progression to malignancy in the polyoma middle T oncoprotein mouse breast cancer model provides a reliable model for human diseases.. Am J Pathol.

[24] Aguirre AJ, Bardeesy N, Sinha M, Lopez L, Tuveson DA (2003). Activated Kras and Ink4a/Arf deficiency cooperate to produce metastatic pancreatic ductal adenocarcinoma.. Genes Dev.

[25] Rauch A, Bellew M, Eng J, Fitzgibbon M, Holzman T (2006). Computational Proteomics Analysis System (CPAS): an extensible, open-source analytic system for evaluating and publishing proteomic data and high throughput biological experiments.. J Proteome Res.

[26] Craig R, Beavis RC (2004). TANDEM: matching proteins with tandem mass spectra.. Bioinformatics.

[27] Maclean B, Eng JK, Beavis RC, McIntosh M (2006). General framework for developing and evaluating database scoring algorithms using the TANDEM search engine.. Bioinformatics.

[28] Keller A, Nesvizhskii AI, Kolker E, Aebersold R (2002). Empirical statistical model to estimate the accuracy of peptide identifications made by MS/MS and database search.. Anal Chem.

[29] Faca V, Coram M, Phanstiel D, Glukhova V, Zhang Q (2006). Quantitative analysis of acrylamide labeled serum proteins by LC-MS/MS.. J Proteome Res.

[30] Kersey PJ, Duarte J, Williams A, Karavidopoulou Y, Birney E (2004). The International Protein Index: an integrated database for proteomics experiments.. Proteomics.

[31] Gabay C, Kushner I (1999). Acute-phase proteins and other systemic responses to inflammation.. N Engl J Med.

[32] Girardi G, Yarilin D, Thurman JM, Holers VM, Salmon JE (2006). Complement activation induces dysregulation of angiogenic factors and causes fetal loss.. American Journal of Reproductive Immunology.

[33] Carmeliet P (2001). Biomedicine. Clotting factors build blood vessels.. Science.

[34] Cid MC, Grant DS, Hoffman GS, Auerbach R, Fauci AS (1993). Identification of haptoglobin as an angiogenic factor in sera from patients with systemic vasculitis.. J Clin Invest.

[35] Alam R, Forsythe P, Stafford S, Heinrich J, Bravo R (1994). Monocyte chemotactic protein-2, monocyte chemotactic protein-3, and fibroblast-induced cytokine. Three new chemokines induce chemotaxis and activation of basophils.. J Immunol.

[36] Yang W, Hood BL, Chadwick SL, Liu S, Watkins SC (2008). Fatty acid synthase is up-regulated during hepatitis C virus infection and regulates hepatitis C virus entry and production.. Hepatology.

[37] ten Dijke P, Arthur HM (2007). Extracellular control of TGFbeta signalling in vascular development and disease.. Nat Rev Mol Cell Biol.

[38] Blokzijl A, Dahlqvist C, Reissmann E, Falk A, Moliner A (2003). Cross-talk between the Notch and TGF-beta signaling pathways mediated by interaction of the Notch intracellular domain with Smad3.. J Cell Biol.

[39] Fernandez CA, Yan L, Louis G, Yang J, Kutok JL (2005). The matrix metalloproteinase-9/neutrophil gelatinase-associated lipocalin complex plays a role in breast tumor growth and is present in the urine of breast cancer patients.. Clin Cancer Res.

[40] Yang J, Bielenberg DR, Rodig SJ, Doiron R, Clifton MC (2009). Lipocalin 2 promotes breast cancer progression.. Proc Natl Acad Sci U S A.

[41] Ye B, Cramer DW, Skates SJ, Gygi SP, Pratomo V (2003). Haptoglobin-alpha subunit as potential serum biomarker in ovarian cancer: identification and characterization using proteomic profiling and mass spectrometry.. Clin Cancer Res.

[42] Erler JT, Bennewith KL, Nicolau M, Dornhofer N, Kong C (2006). Lysyl oxidase is essential for hypoxia-induced metastasis.. Nature.

[43] Chan JM, Stampfer MJ, Giovannucci E, Gann PH, Ma J (1998). Plasma insulin-like growth factor-I and prostate cancer risk: a prospective study.. Science.

[44] Akkiprik M, Feng Y, Wang H, Chen K, Hu L (2008). Multifunctional roles of insulin-like growth factor binding protein 5 in breast cancer.. Breast Cancer Res.

[45] Yee KO, Connolly CM, Duquette M, Kazerounian S, Washington R (2009). The effect of thrombospondin-1 on breast cancer metastasis.. Breast Cancer Res Treat.

[46] Yi CH, Smith DJ, West WW, Hollingsworth MA (2007). Loss of fibulin-2 expression is associated with breast cancer progression.. Am J Pathol.

[47] Prenzel KL, Warnecke-Eberz U, Xi H, Brabender J, Baldus SE (2006). Significant overexpression of SPARC/osteonectin mRNA in pancreatic cancer compared to cancer of the papilla of Vater.. Oncol Rep.

[48] Wong K, Park HT, Wu JY, Rao Y (2002). Slit proteins: molecular guidance cues for cells ranging from neurons to leukocytes.. Curr Opin Genet Dev.

[49] Feldmann M, Brennan FM, Maini RN (1996). Role of cytokines in rheumatoid arthritis.. Annu Rev Immunol.

[50] Jakowlew SB (2006). Transforming growth factor-beta in cancer and metastasis.. Cancer Metastasis Rev.

[51] Chen W, Jin W, Cook M, Weiner HL, Wahl SM (1998). Oral delivery of group A streptococcal cell walls augments circulating TGF-beta and suppresses streptococcal cell wall arthritis.. J Immunol.

[52] Dajee M, Lazarov M, Zhang JY, Cai T, Green CL (2003). NF-kappaB blockade and oncogenic Ras trigger invasive human epidermal neoplasia.. Nature.

