# Supplementary material for: Plasma Proteome Profiles Associated with Inflammation, Angiogenesis, and Cancer
Source: PLoS One. 2011 May 12;6(5):e19721. doi: 10.1371/journal.pone.0019721 (PMC3093388; doi:10.1371/journal.pone.0019721)

Supplementary Figure 1A: Acute Inflammation

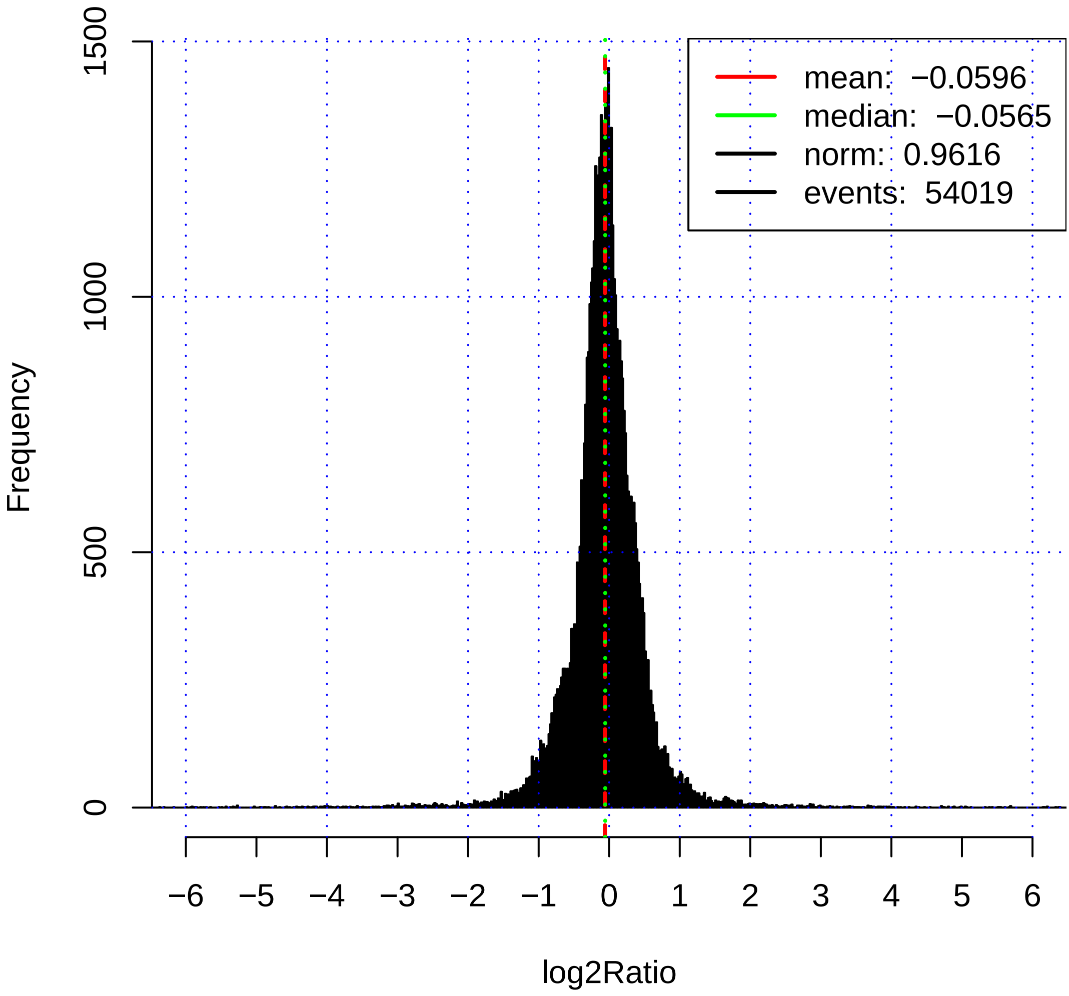

Supplementary Figure 1B: Chronic Inflammation

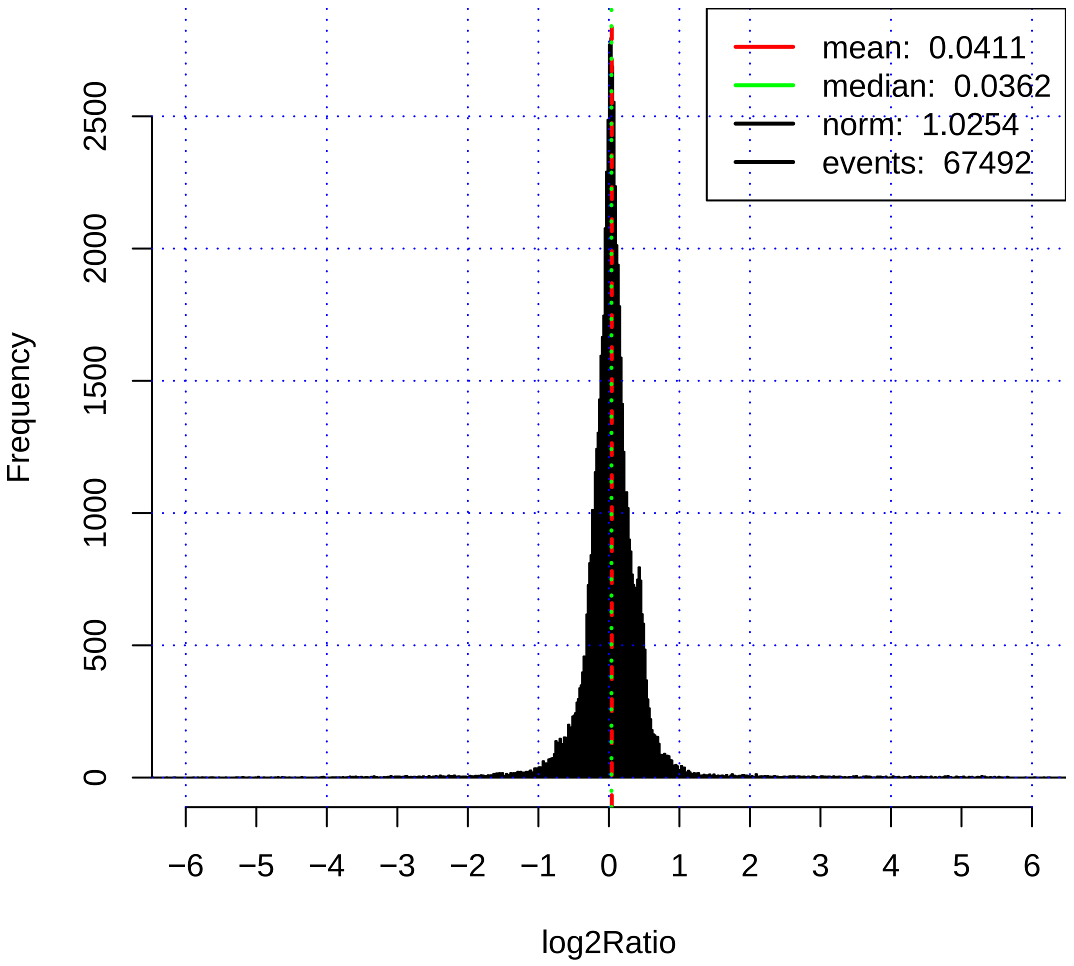

Supplementary Figure 1C: Angiogenesis

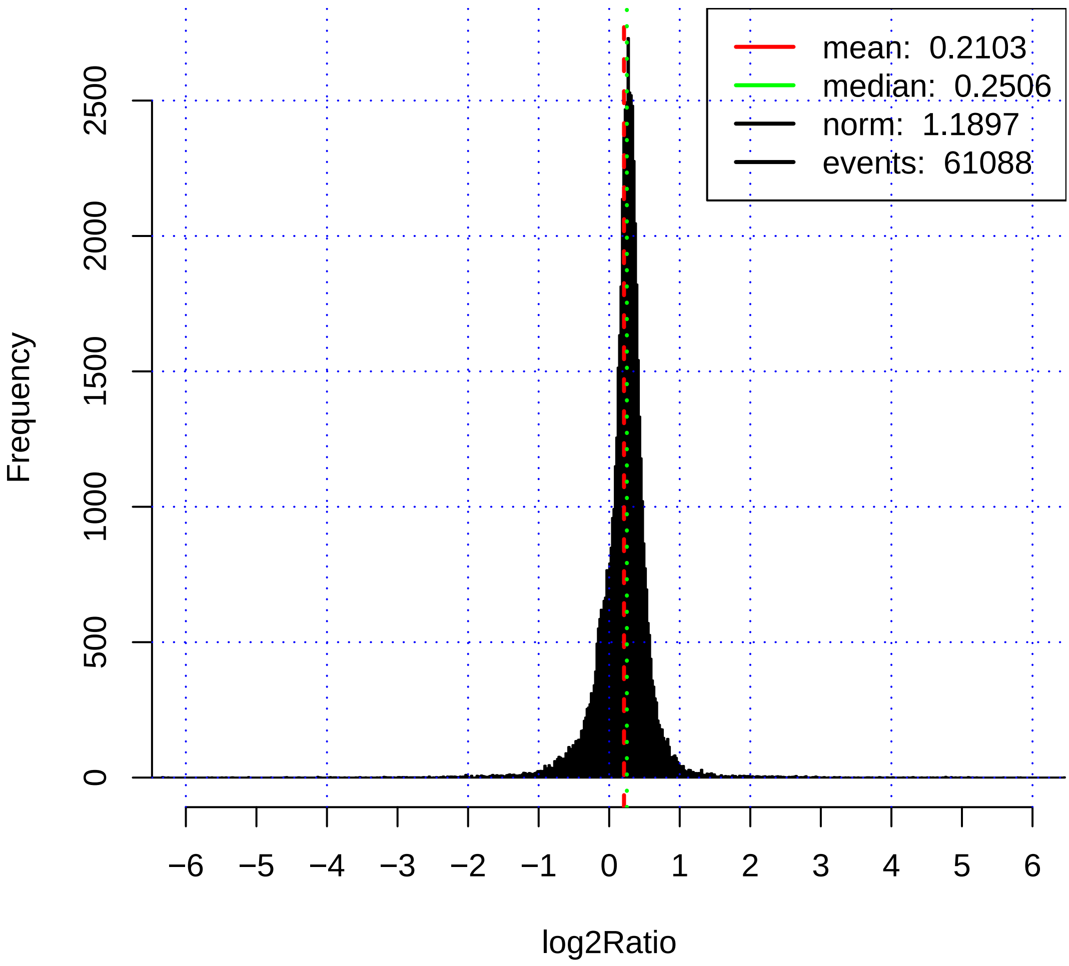

Supplement: Figure S1 — Histograms of case/control peptide ratios. A) acute inflammation, B) chronic inflammation, and C) angiogenesis. (PDF) [file pone.0019721.s001.pdf]
